# Dynamic Changes of the Infralimbic Cortex and Its Regulation of the Prelimbic Cortex in Rats with Chronic Inflammatory Pain

**DOI:** 10.1007/s12264-023-01159-x

**Published:** 2024-01-05

**Authors:** Longyu Ma, Lupeng Yue, Shuting Liu, Yu Zhang, Meng Zhang, Shuang Cui, Feng-Yu Liu, Ming Yi, You Wan

**Affiliations:** 1 Department of Neurobiology, School of Basic Medical Sciences, Neuroscience Research Institute, Peking University, Beijing, 100083 China; 2 CAS Key Laboratory of Mental Health, Institute of Psychology, Beijing, 100101 China; 3 Department of Psychology, University of Chinese Academy of Science, Beijing, 100101 China; 4 NHC Key Laboratory of Human Disease Comparative Medicine, Institute of Laboratory Animal Sciences, CAMS & PUMC, Beijing, 100021 China; 5 Department of Pathology, Beijing Children’s Hospital, Capital Medical University, National Center for Children’s Health, Beijing, 100045 China; 6 Key Laboratory for Neuroscience, Ministry of Education/National Health Commission, Peking University, Beijing, 100083 China; 7 Co-innovation Center of Neuroregeneration, Nantong University, Nantong, 226001 China

**Keywords:** Prefrontal cortex, Prelimbic cortex, Infralimbic cortex, Inhibitory regulation, Chronic pain

## Abstract

The prelimbic cortex (PL) is actively engaged in pain modulation. The infralimbic cortex (IL) has been reported to regulate the PL. However, how this regulation affects pain remains unclear. In the present study, we recorded temporary hyper-activity of PL pyramidal neurons responding to nociceptive stimuli, but a temporary hypo-function of the IL by *in vivo* electrophysiological recording in rats with peripheral inflammation. Manipulation of the PL or IL had opposite effects on thermal hyperalgesia. Furthermore, the functional connectivity and chemogenetic regulation between the subregions indicated an inhibitory influence of the IL on the PL. Activation of the pathway from the IL to the PL alleviated thermal hyperalgesia, whereas its inhibition exacerbated chronic pain. Overall, our results suggest a new mechanism underlying the role of the medial prefrontal cortex in chronic pain: hypo-function of the IL leads to hyperactivity of the PL, which regulates thermal hyperalgesia, and thus contributes to the chronicity of pain.

## Introduction

Pain is one of the most common clinical symptoms. Acute pain acts as a warning signal, whereas chronic pain is associated with numerous long-term deleterious outcomes including depression, insomnia, cognitive impairment, and maladaptive stress responses [1, 2]. In the development of chronic pain, the prefrontal cortex (PFC) shows substantial morphological [3, 4], electrophysiological [5, 6], and functional [7] changes. Accumulating evidence from patients and model animals has revealed that the PFC participates in pain modulation: functional connectivity between and nucleus accumbens predicts the transition from acute to chronic pain [8], whereas PFC-dependent cognitive capacities predict the occurrence of post-operational pain [9].

The prelimbic cortex (PL) is a core medial PFC subregion in rodents that mediates chronic pain. Increased neuronal activity in human dorsolateral PFC (a prefrontal area related by some studies to the rodent PL) is correlated with pain intensity in chronic back pain [10]. Furthermore, a nociceptive neuronal ensemble that contributes to pain chronicity has been identified in the mouse PL [11]. More intriguingly, functional alterations in the PL persist even after the perceptual recovery from chronic pain and facilitate nociceptive responses to subsequent noxious exposure [12], suggesting that upstream inputs may suppress PL activity and cooperatively determine pain expression. The infralimbic cortex (IL), another subregion of the rodent medial PFC, appears to be tuned to the regulation of nociception and is one such candidate [7, 13]. Specifically, there is a direct connection between the IL and the PL: excitatory pyramidal neurons in the deep layers of the IL innervate inhibitory interneurons in the deep layers of the PL, and these further synapse onto local pyramidal neurons [5, 14]. This pathway has been implicated in the learning of alternative associations, which depends on the direct connectivity from IL to PL [15]. In rodents, the excitability of layer 5 pyramidal neurons in the IL decreases in complete Freund’s adjuvant (CFA)-induced inflammation in mice and appears to inhibit connectivity with other brain regions in chronic pain [16, 17]. Taken together, previous studies have suggested that the rodent IL might provide crucial modulatory afferents for the PL, and have complementary and perhaps opposite roles in the processing of nociception in chronic pain. However, the direct comparison of their functions and encoding patterns during nociceptive responses is still lacking.

In the present study, we used *in vivo* electrophysiological recordings, nuclear lesions, chemogenetics methods, and behavioral tests to investigate how the PL and IL encode nociceptive information and modulate the development of chronic pain. Taken together, our results define distinct and opposite roles of the PL and the IL specifically to mediate nociceptive responses and regulate pain behavior, and provide circuit-level evidence that the inhibitory regulation from the IL to the PL in the development of thermal hyperalgesia in different phases of chronic inflammatory pain induced by intraplantar CFA injection in rats.

## Materials and Methods

### Experimental Animals

Male Sprague-Dawley rats (200–250 g for lesion and chemogenetic experiments, and 250–300 g for multichannel recording experiments) were supplied by the Experimental Animal Center of Peking University Health Science Center. To avoid damage to cannulas and microdrives, the rats were housed individually at a constant temperature of 23 ℃ under a 12 h light/dark cycle with food and water available *ad libitum*. All experimental procedures complied with the Guidelines of the Animal Care and Use Committee of our University (approval number: LA2021144). Rats were handled for at least three days before any experiments.

### Establishment of the CFA-Induced Inflammatory Pain Model

100 µL of CFA (Sigma-Aldrich, St Louis, USA) was intraplantarly injected into the left hind paw to induce inflammatory pain. The same volume of normal saline was injected as a control. For 10% CFA injection, CFA was diluted 10-fold in volume with normal saline, and 100 μL 10% CFA was injected into the rat's hind paw to induce mild inflammation.

### Behavioral Test

Before testing, the animals were allowed to acclimatize to the experimental environment for 20 min. The power of the radiant heat source was adjusted to obtain average baseline paw withdrawal latencies (PWLs) of 15–18 s, with a cut-off time of 30 s to prevent any possible tissue damage. PWLs were measured three times at 5-min intervals, and the mean value was used to reflect the level of thermal hyperalgesia. Behavioral testing was applied in a single-blinded manner.

### Stereotaxic Surgery

Rats were anesthetized with 1% pentobarbital sodium (0.1 g/kg, *i.p.*) and positioned in a stereotaxic instrument (RWD, Shenzhen, China). For PL or IL lesion experiments, 0.5 μL of 0.09 mol/L quinolinic acid (QA, Sigma-Aldrich) in phosphate-buffered saline (pH 7.4) was administered through a 2-μL Hamilton microsyringe in 5 min (0.1 μL/min/side) to the bilateral PL or IL according to the atlas of Paxinos and Watson (1997) PL: AP +3.5, ML ±0.5, DV –2.5; IL: AP +3.0, ML ±1.18, DV –4.56 mm, angle (ML-DV plane, away from mid-line) 15°. The coordinates are relative to the Bregma. All injections were followed by an additional 5 min to allow drug diffusion before removal of the injection needle.

For cannula implantation and chemogenetic virus injection, to avoid laterality, we manipulated both lateral cortices and neural pathways simultaneously. Stainless steel, 22-gauge guide cannulas were bilaterally implanted 1 mm above the target site as described above. The cannulas were secured to screws with dental acrylic on the skull. For retrograde labeling of IL→PL connectivity, the retroAAV2-hSyn-Cre (AAV2-Retro) (0.5 μL/side, 1.0 × 10^13^ virus particles/ml, OBIO Technology, Shanghai, China) was injected into the bilateral PL. AAV2-CaMKII-DIO-hM3Dq/hM4Di-mCherry (0.5 μL/side, 1.4 × 10^13^ virus particles/ml, OBIO Technology, Shanghai, China) was injected into the bilateral IL as described above. Electrophysiological recordings were made 3 weeks after the virus injection. For behavioral tests, in the experiments of manipulating the PL or IL, AAV5-CaMKII-hM3Dq-mCherry, AAV5-CaMKII-hM4Di-mCherry, and AAV5-CaMKII-mCherry (1.4 × 10^13^ virus particles/ml) were provided as gifts by Dr. Daniel J. Urben’s Lab (University of North Carolina, USA). Virus solution of 0.5 μL/side was stereotaxically injected into the PL or the IL bilaterally as described above. Stainless steel stylets were placed in the guide cannulas to prevent clogging. In the experiments manipulating the IL–PL pathway, AAV5-CaMKII-hM3Dq-mCherry, AAV5-CaMKII-hM4Di-mCherry, and AAV5-CaMKII-mCherry (0.5 μL/side, 1.4 × 10^13^ virus particles/ml, University of North Carolina, USA) were injected into the IL and the cannulas were implanted in the PL bilaterally. Behavioral tests were started three weeks later to ensure virus expression. Clozapine N-oxide (CNO; Tocris, Bristol, UK) was dissolved in artificial cerebrospinal fluid to 0.8 mmol/L, and 0.5 μL was administered per side based on previous studies [18].

For *in vivo* multichannel recording, hand-made 8 tetrodes were implanted for simultaneous PL (AP +3.5 to 4.0, ML +0.5, DV –2.5) and IL (IL: AP + 2.5 to 3.5, ML +0.5, DV –4.4 mm, relative to Bregma) recordings in each rat. After surgery, the rat was returned to its home cage and allowed to recover for one week before electrophysiological recordings.

### *In vivo* Electrophysiological Recordings

After recovery from surgery, the tetrodes were slowly lowered to screen for spikes. After reaching a suitable position, the electrodes were stabilized without further movement.

Each rat was allowed to move freely in a transparent plastic chamber (30 cm × 30 cm × 40 cm) with video recording. The chamber floor was a grid plate with stainless-steel bars 2 mm in diameter and 8 mm in between. Electrophysiology data were acquired using a 32-channel Intan system (Intan Technologies, Los Angeles, USA). An electrode interface board was connected to the head stage, which was connected to the system amplifier with cables. After habituation in the environment, the spontaneous neural activities were obtained from the free-moving rats for 20 min. Another group of rats was used for recording the evoked neuronal response. The nociceptive stimuli were 20 noxious laser stimuli and 20 von Frey hair stimuli. Laser stimulation was generated by an ultra-pulse carbon dioxide laser therapeutic machine (DM-300, Dimei, Changchun, China) and delivered to the hind paw of the recorded rat from the guide arm. The tip of the guide arm was kept away from the plantar surface of the paw at a distance of 2 cm. The focus of the laser beam was altered a little from trial to trial to avoid any possible tissue damage. The laser power ranged from 2 to 4 W with an emission time of 20 ms. The power used for each rat was the lowest power to induce >8 trials with paw lifting behaviors in 10 laser stimuli, determined by a pilot experiment before formal recording. A 15-g von Frey hair was applied to the central plantar surface of the hind paw to induce a mechanical nociceptive response. The interstimulus interval was no less than 60 s to avoid hyperalgesia. For electrophysiological verification of DREADDs (Designer Receptor Exclusively Activated by Designer Drugs), rats were anesthetized and implanted with an electrode with an adjacent cannula. The spikes and local field potentials (LFPs) were recorded and analyzed between 10 min before and 20 min after CNO infusion.

### Electrophysiological Data Analysis

#### Data Preprocessing and Spike Sorting

Electrophysiological data were preprocessed using NDManager and customized MatLab scripts. To obtain the LFPs, the data were down-sampled to 1,250 Hz, bandpass filtered between 1 and 100 Hz, and notch filtered between 48 and 52 Hz.

Spike sorting was carried out in KlustaKwik (http://klustakwik.sourceforge.net/). Using principal component analysis, a rough separation of units from pyramidal and interneurons was mainly based on their differences in spike wave-shapes. Putative interneurons had a narrow peak-to-valley width (<0.5 ms), and putative pyramidal neurons showed a wide peak-to-valley width (>0.5 ms) [19]. The spikes with low firing rates (<0.5 Hz) were excluded from further analysis.

#### Power Spectral Estimation

Power spectral analysis was applied using Chronux (http://chronux.org/). The bandwidth product was 3 and 5 tapers were used. LFP signals were fragmented into 10-s segments and filtered at a bandpass of 1−46 Hz to avoid motor and mechanical noise. The relative power spectral density (PSD) at each frequency was calculated as the percentage of total PSDs within the 1–46 Hz frequency range. The PSD was divided into 5 frequency bands: 1−4 Hz (delta), 4–8 Hz (theta), 8–13 Hz (alpha), 13–30 Hz (beta), and 30–46 Hz (gamma).

Subsequently, we applied point-by-point t-test analysis to identify the frequency intervals in which the differences of relative PSD between the CFA and sham groups. The significance level (expressed as *P* value) was corrected using a false discovery rate (FDR) procedure.

#### Neuronal Responses to External Stimuli

To measure neuronal responses to external stimuli, the firing rates of spike units were calculated for the period between 2 s before and 2 s after the stimulus onset in each trial. The spike trains were binned using a 100-ms window. To classify whether a unit showed a significant response to the external stimuli, the paired *t* test was applied to compare the firing rate of each bin and the binned averaged firing rate of baseline (the averaged firing rate from the bins the 2 s before the stimulus onset) among stimuli trials. The spike units were divided into excitatory or inhibitory groups if they showed any significant increase or decrease in firing rate compared to the baseline in any bin after the stimulus onset. To evaluate the extent of firing variation, we used the modulation index which was defined as follows:

$$\mathrm{index}=\left|\frac{{F}_{\mathrm{after}}-{F}_{\mathrm{before}}}{{F}_{\mathrm{after}}+{F}_{\mathrm{before}}}\right|$$

*F*_before_ and *F*_after_ were the averaged firing rates for 2 s before and 2 s after stimulus onset, respectively.

#### Generalized Partial Directed Coherence Analysis

Generalized partial directed coherence (gPDC) was calculated as previously described [20–22]. This algorithm was based on a multi-variate autoregressive (MVAR) model, which integrated both PL and IL LFP signals.

In brief, the two different time series were modeled as a form:

$$\left[\begin{array}{c}{x}_{1}(t)\\ {x}_{2}(t)\end{array}\right]={\sum }_{r=1}^{p}Ar\left(\left[\begin{array}{c}{x}_{1}\left(t-r\right)\\ {x}_{2}\left(t-r\right)\end{array}\right]\right)+ \left[\begin{array}{c}{u}_{1}(t)\\ {u}_{2}(t)\end{array}\right]$$

[(u1(t), u2(t)] represents uncorrelated Gaussian white noise processes representing the model residuals, the model order p was determined by the minimum of the Akaike Information Criterion or Bayesian Information Criterion. The gPDC was defined as follows:

$$\left|{C}_{i\leftarrow j}(f)\right| = \frac{{a}_{ij}(f)}{\sqrt{{\sum }_{k}{\frac{1}{{{\sigma }_{k}}^{2}}\left|{a}_{kj}(f)\right|}^{2}}}$$

Aij($\mathrm{f}$) is the Fourier transformation of the MVAR coefficient ($$\mathrm{Ar }= \left[\begin{array}{cc}{\mathrm{a}}_{11}& {\mathrm{a}}_{12}\\ {\mathrm{a}}_{21}& {\mathrm{a}}_{22}\end{array}\right]$$), σ_k_ represents the standard deviation of the model residuals. The gPDC value is in the interval of [0 1], where “0” stands for the absence of an influence of a target on the source, and “1” stands for a linearly predictable target from the source.

We also applied point-by-point *t*-test analysis to identify the frequency intervals in which the gPDC values differed between the two groups. The significance level (expressed as *P* value) was corrected using an FDR procedure.

### Histology

Each rat was deeply anesthetized with 1% sodium pentobarbital (0.1 g/kg, *i.p.*) and perfused with 4% paraformaldehyde (PFA) after all experiments. To confirm the electrode location, the recording sites were marked by a direct current injection (20 μA for 15–20 s) through one microwire per array before PFA perfusion. The whole brain was removed after perfusion, post-fixed in 4% PFA overnight, stored in 30% sucrose, and cut into 30-μm frozen sections for immunohistochemical staining. The sections were Nissl counterstained to help visualize the electrode tracks and lesion sites under a light microscope. Virus infection was confirmed after behavioral tests by detecting fluorescein using a fluorescence microscope (Leica DMI 4000B, Wetzlar, Germany).

### Statistical Analysis

Data are presented as the mean ± SEM. Group comparisons were made using either one-way or two-way analysis of variance (ANOVA) followed by Bonferroni post hoc tests. Single variable comparisons were made with Student’s *t* test. χ^2^ analyses or Fisher’s exact test were used to compare proportions. Mann-Whitney tests or Wilcoxon signed-rank tests were used to compare *in vivo* neural firing rates. The relationship between PSD and recovery rate was assessed using Pearson’s correlation coefficient. The criterion for statistical significance was *P <0.05, and differences were calculated using GraphPad Prism 8.0 and MatLab 2022b.

## Results

### PL and IL Exhibit Different Neuronal Activities in Chronic Inflammatory Pain

**Fig. 1:**
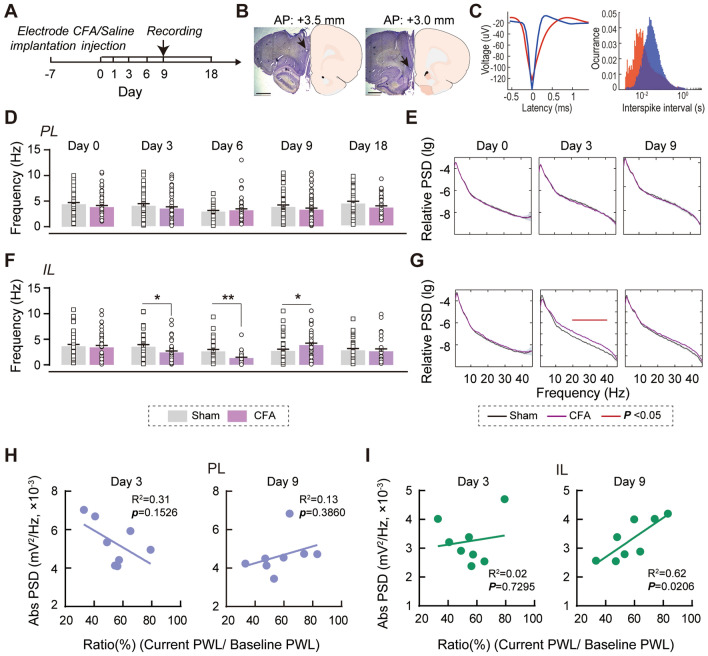
Temporary hypo-activity of IL neurons in the sub-acute phase of inflammatory pain. **A** Diagram showing the recording procedure. **B** Representative electrode track in the PL (left) and the IL (right). Scale bars, 1.25 mm. **C** Representative waveform (left) and ISI (right) of spikes yielding two single units; red, a putative pyramidal neuron; blue, a putative interneuron. **D, E** No significant difference in spontaneous firing rates (**D**) and relative power spectral densities (PSDs) (**E**) at different time points during inflammatory pain in the PL. Data from 8 rats per group. Mann-Whitney *U* test. **F, G** Decreased spontaneous firing rates on CFA days 3 and 6, increased spontaneous firing rates on CFA day 9, and lower PSDs in the beta and gamma bands (17−42 Hz) on CFA day 3 in the IL. Red line, the frequency range with statistically significant differences. Data from 8 rats per group. *P <0.05, **P <0.01, Mann-Whitney *U* test. **H** No significant correlations on both CFA day 3 and day 9 in the PL. *n* = 8 per group. Pearson’s correlation test. **I** The absolute PSDs in beta and gamma bands are positively correlated with the extent of hyperalgesia alleviation on CFA day 9, (*P* = 0.02, Pearson correlation) but not on CFA day 3 in the IL. *n* = 8 per group. Pearson’s correlation test.

To reveal the functional changes of the PFC in rats with the development of chronic inflammatory pain, we first made electrophysiological recordings in layer V, a layer mainly composed of large pyramidal neurons in the PL and the IL of freely-moving rats with CFA-induced inflammatory pain (Fig. 1A, B). Among these recordings, 1,481 were from putative pyramidal neurons, based on their waveform and firing properties (Fig. 1C). Putative interneurons were excluded from further analysis considering their low proportion (245 units).

In the resting state, we found that IL neurons showed decreased spontaneous firing on CFA days 3–6, but increased firing on CFA day 9 (3 days: u = 668, *P* = 0.0394; 6 days: u = 401.5, *P* = 0.0096; 9 days: u = 709, *P* = 0.0113, Mann-Whitney *U* test; Fig. 1F), and lower relative PSDs in the beta and gamma bands (17−42 Hz) (Fig. 1G). By contrast, the PL showed no changes in the spontaneous firing rate (Fig. 1D) and PSD (Fig. 1E) after CFA injection. Moreover, we found a positive correlation between absolute PSDs in the beta and gamma bands and the recovery rate from hyperalgesia on CFA day 9 in the IL (R^2^ = 0.62, *P* = 0.0206, Pearson’s correlation test; Fig. 1I), but not in the PL (Fig. 1H), suggesting that suppressed activity in the IL and recovery of the IL from hypo-function could alleviate hyperalgesia.

**Fig. 2:**
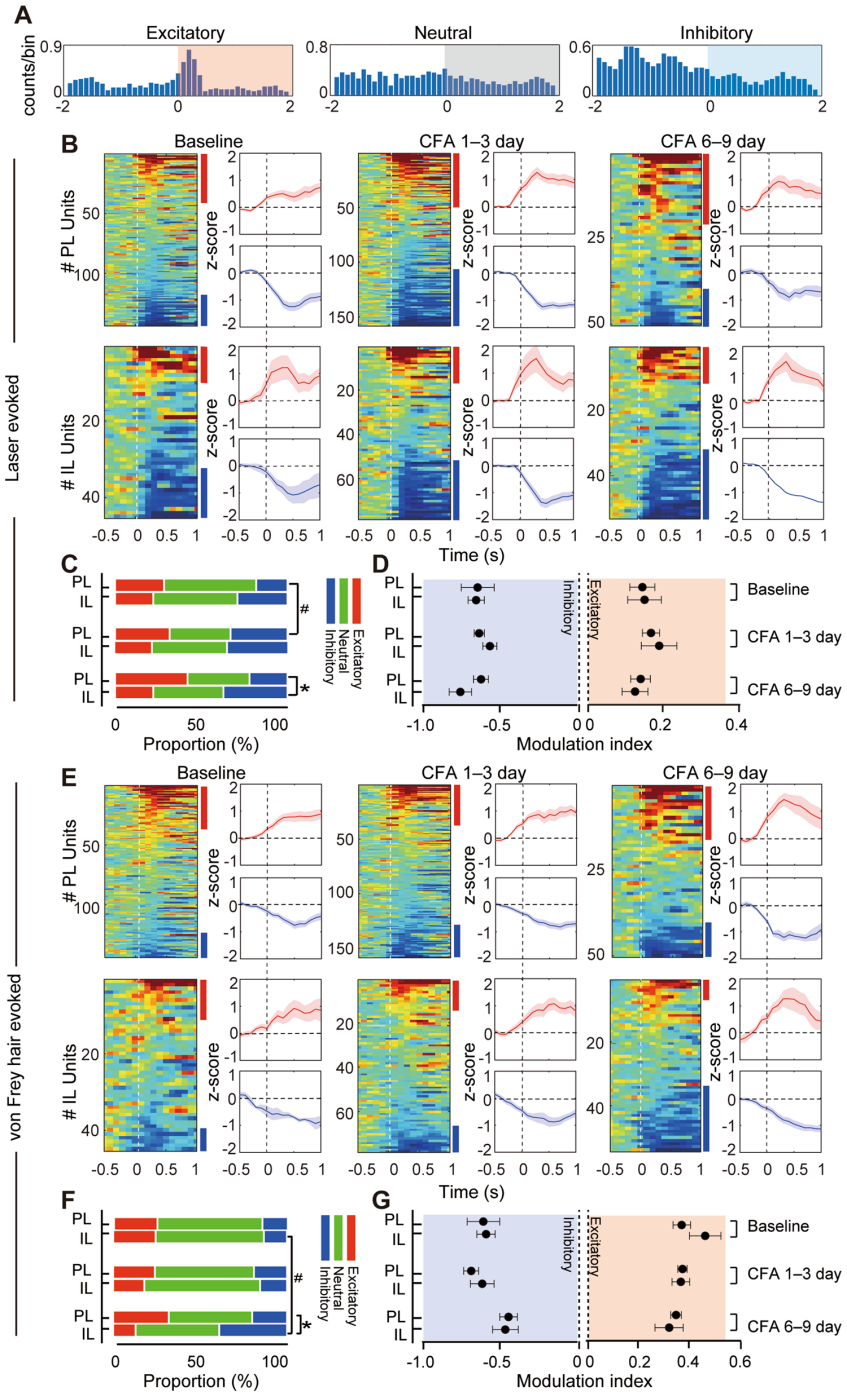
Different nociceptive responses to evoked stimuli of the PL and IL in chronic inflammatory pain. **A** Representative excitatory (left), neutral (middle), and inhibitory (right) neuronal response to nociceptive stimuli. Peri-stimulus time histograms (PSTHs) show the average firing count across all trials, relative to stimulus onset (100-ms bins). **B** Laser-evoked responses of PL (upper panel) and IL (lower panel) neurons at baseline (day 0), CFA 1–3 days, and CFA 6–9 days after modeling. Heatmap rows represent the Z score-transformed average PSTH for individual neurons, and columns represent time bins relative to laser onset (50 ms in width). Blue and red bars indicate statistically significant laser-responsive units. Plots to the right show the average Z score responses for laser-excitatory (upper) and laser-inhibitory units (lower). Shaded area, SEM. **C** A larger proportion of PL neurons show excitatory nociceptive responses to the laser during 6–9 days after CFA injection. Data from 9 rats per group. *P <0.05 *vs* IL, ^#^*P* <0.05 *vs* Baseline, χ^2^ test. **D** The amplitude of laser-evoked responses in PL and IL neurons remain similar in inflammatory pain. Two-way ANOVA. **E** von Frey hair-evoked responses of PL (upper panel) and IL (lower panel) neurons at baseline (day 0), CFA 1–3 days, and CFA 6–9 days after modeling. **F** A larger proportion of PL neurons show excitatory nociceptive responses to von Frey hair during 6–9 days after CFA injection. Data from 9 rats per group. *P <0.05 *vs* IL, ^#^*P* <0.05 *vs* Baseline, χ^2^ test. **G** The amplitude of von Frey hair-evoked responses in PL and IL neurons remains similar in inflammatory pain. Two-way ANOVA.

To further examine nociceptive responses of prefrontal neurons, we applied two types of nociceptive stimuli to the affected paw (Fig. 2A). For the thermal nociceptive stimulus (laser) (Fig. 2B), we found a remarkably increased proportion of laser-excitatory neurons in the PL and a decreased proportion of laser-inhibitory neurons in the IL during 6–9 days after CFA injection. The proportion of excitatory responsive neurons in the PL also increased during days 1–3 when compared with baseline (comparing the PL and IL groups, Baseline: *χ*^2^_(2)_ = 2.584, *P* = 0.2724; days 1–3: *χ*^2^_(2)_ = 2.739, *P* = 0.2524; days 6–9: *χ*^2^_(2)_ = 6.530, *P* = 0.0382; comparing Baseline and after modeling, PL: Baseline *vs* days 1–3: *χ*^2^_(2)_ = 12.25, *P* = 0.0022; Baseline *vs* days 6–9: *χ*^2^_(2)_ = 2.991, *P* = 0.2241. IL: Baseline *vs* days 1–3: *χ*^2^_(2)_ = 0.4661, *P* = 0.7921; Baseline *vs* days 6–9:* χ*^2^_(2)_ = 0.8556, *P* = 0.6520; Fig. 2C). The magnitude of neuronal responses remained similar across all phases (excitatory: time effect: *F*_(2,145)_ = 0.8557, *P* = 0.4271; group effect: *F*_(1,145)_ = 0.0191, *P* = 0.8902; interaction: *F*_(2,145)_ = 10.1237, *P* = 0.8837; inhibitory: time effect: *F*_(2,145)_ = 1.562, *P* = 0.2132; group effect: *F*_(1,145)_ = 0.2860, *P* = 0.5936; interaction: *F*_(2,145)_ = 1.912, *P* = 0.1515, two-way ANOVA; Fig. 2D). For the mechanical nociceptive stimulus (von Frey hair), the changes of proportion and magnitude of neuronal responses were equivalent to the laser-evoked responses. The proportion of inhibitory responsive neurons in the IL increased during days 6–9 when compared with baseline (comparing the PL and IL groups, Baseline: *χ*^2^_(2)_ = 0.0601, *P* = 0.9704; days 1–3: *χ*^2^_(2)_ = 2.081, *P* = 0.3533; days 6–9: *χ*^2^_(2)_ = 7.362, *P* = 0.0252; comparing Baseline and after modeling, PL: Baseline *vs* days 1–3: *χ*^2^_(2)_ = 1.046, *P* = 0.5926; Baseline *vs* days 6–9: *χ*^2^_(2)_ = 2.360, *P* = 0.3073. IL: Baseline *vs* days 1–3: *χ*^2^_(2)_ = 0.7611, *P* = 0.6835; Baseline *vs* days 6–9: *χ*^2^_(2)_ = 8.549, *P* = 0.0139; excitatory: time effect: *F*_(2,116)_ = 2.428, *P* = 0.0927; group effect: *F*_(1,116)_ = 0.4653, *P* = 0.4965; interaction: *F*_(2,116)_ = 1.350, *P* = 0.2632; inhibitory: time effect: *F*_(2,94)_ = 3.209, *P* = 0.0449; group effect: *F*_(1,94)_ = 0.1321, *P* = 0.7171; interaction: *F*_(2,94)_ = 0.1869, *P* = 0.8298, two-way ANOVA; Fig. 2E–G).

These results show distinct PL and IL neuronal properties in inflammatory pain. Temporary hypo-activity in the IL paralleled the stronger excitatory responses in the PL to the nociceptive stimuli 3–6 days after CFA injection, and the spontaneous recovery from IL hypo-activity was associated with the alleviation of thermal hyperalgesia.

### The PL and IL Contribute in Opposite Directions to the Maintenance of Thermal Hyperalgesia in Chronic Inflammatory Pain

**Fig. 3:**
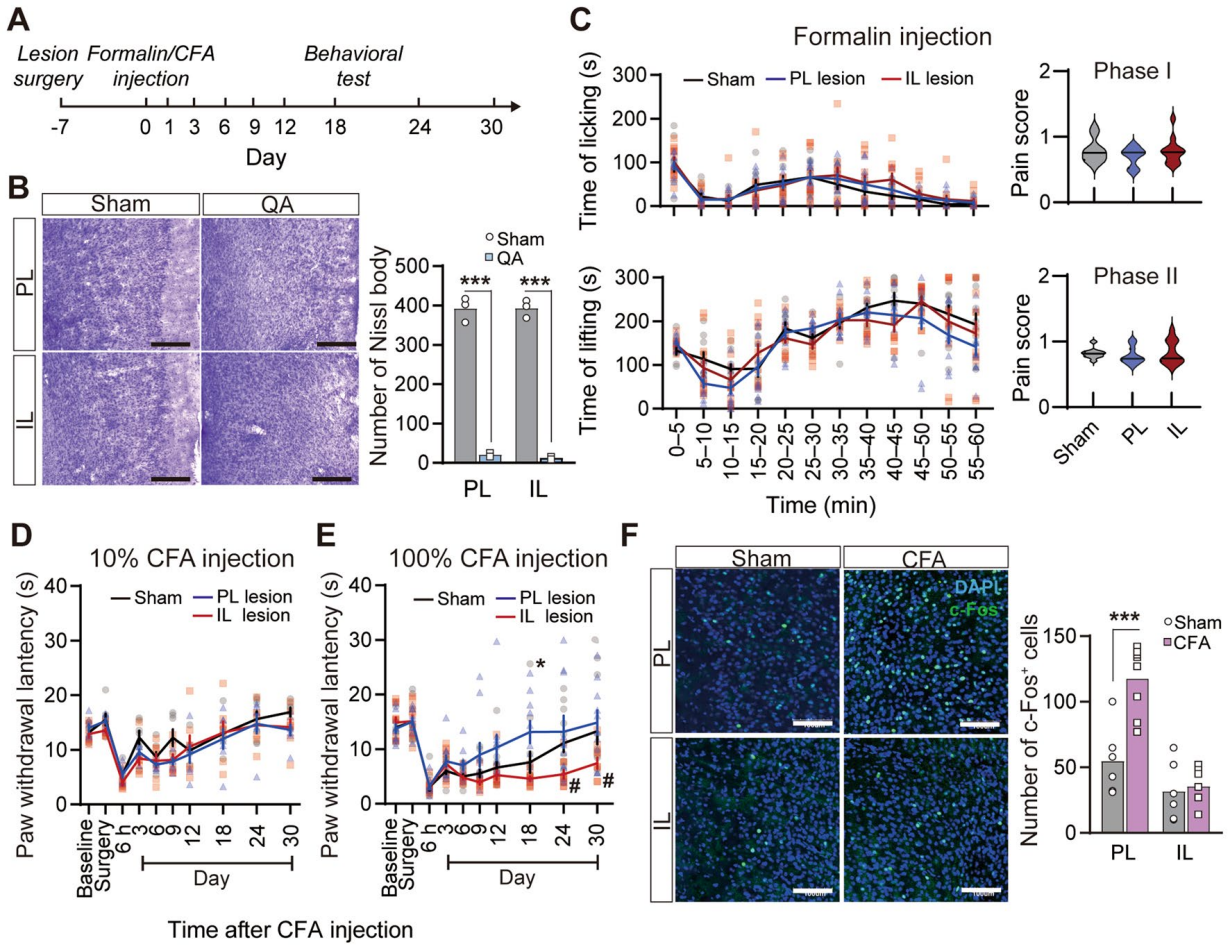
Opposite effects on the development of thermal hyperalgesia after bilateral lesions of the IL and PL in chronic inflammatory pain. **A** Diagram showing the timeline of experiments. Rats received intraplantar injection of formalin or CFA or saline 7 days after the PL or IL lesions. PWLs in response to a thermal stimulus on days 1, 3, 6, 9, 12, 18, 24, and 30 after intraplantar CFA or saline injection. Formalin pain behaviors were measured 1 h after formalin injection. **B** Representative images showing Nissl bodies in the PL and the IL after QA lesions. Scale bars, 200 μm. QA lesions decreased the number of Nissl bodies in the PL and IL regions. *n* = 3 per group. ***P <0.001, unpaired *t* test. **C** Formalin-induced acute pain behaviors are not affected by lesions of the PL or IL.* n* = 11–12 per group. Two-way ANOVA with repeated measures. **D** 10% CFA-induced mild inflammatory pain behaviors are not affected by lesions of the PL or IL. *n* = 7 per group. Two-way ANOVA with repeated measures. **E** Lesions of the PL attenuate, but lesions of the IL aggravate thermal hyperalgesia in CFA-induced inflammatory pain. *n* = 10–11 per group. *P <0.05, PL lesion *vs* Sham, ^#^*P* <0.05, IL lesion *vs* Sham. Two-way ANOVA with repeated measures and Bonferroni’s *post hoc* test. **F** Representative immunofluorescence images showing c-Fos-positive neurons in the PL and IL of rats with CFA or saline injection. Scale bars, 100 μm. CFA induces robust c-Fos expression in the PL.* n* = 3 per group. ***P <0.001, unpaired *t* test.

To determine the function of the PFC in pain modulation, we made bilateral PL or IL lesions with QA in three pain models (Fig. 3A). Compared with the sham group, injection of QA significantly reduced the amount of Nissl substance, leading the loss of function in the brain regions (PL: *t*_(4)_ = 20.36, *P* <0.001; IL: *t*_(4)_ = 29.04, *P* <0.001, unpaired *t* test; Fig. 3B). The PL or IL lesions had limited effects on formalin-induced nociceptive behaviors (licking: time effect: *F*_(11,330)_ = 26.67, *P* <0.001; group effect: *F*_(2,330)_ = 0.6541, *P* = 0.8825; interaction: *F*_(22,330)_ = 0.6541, *P* = 0.8825; lifting: time effect: *F*_(11,330)_ = 38.08, *P* <0.001; group effect: *F*_(2,330)_ = 1.662, *P* = 0.2067; interaction: *F*_(22,330)_ = 1.510, *P* = 0.0679, two-way ANOVA with repeated measures; phase I: *F*_(2,30)_ = 0.9155, *P* = 0.4112; phase II: *F*_(2,30)_ = 0.3861, *P* = 0.6831, one-way ANOVA; Fig. 3C) and baseline thermal pain thresholds, as well as mild inflammation-induced thermal hyperalgesia (time effect: *F*_(9,162)_ = 32.58, *P* <0.001; group effect: *F*_(2,162)_ = 1.730, *P* = 0.2055; interaction: *F*_(18,162)_ = 1.034, *P* = 0.4255, two-way ANOVA with repeated measures; Fig. 3D). By contrast, lesions of the PL/IL significantly attenuated/aggravated CFA-induced thermal hyperalgesia, mainly in the chronic phase (time effect: *F*_(9,261)_ = 33.61, *P* <0.001; group effect: *F*_(2,261)_ = 5.054, *P* = 0.0131; interaction: *F*_(18,261)_ = 2.790, *P* = 0.0002, two-way ANOVA with repeated measures and Bonferroni’s *post hoc* test; Fig. 3E). Further, we performed c-Fos (a marker for neuronal activity) mapping after thermal stimuli. The expression of c-Fos was sparse in the IL, but the number of c-Fos-positive neurons significantly increased in the PL (PL: *t*_(12)_ = 4.497, *P* = 0.007; IL: *t*_(11)_ = 0.3716, *P* = 0.7137, unpaired *t* test; Fig. 3F), which was consistent with the results for neuronal responses (Fig. 2). These results hint that IL and PL are potentially involved in the process of the maintenance of thermal hyperalgesia in inflammatory pain in opposite directions.

### Genetic Manipulations of PL and IL have Opposite Effects on Thermal Hyperalgesia in Inflammatory Pain

**Fig. 4:**
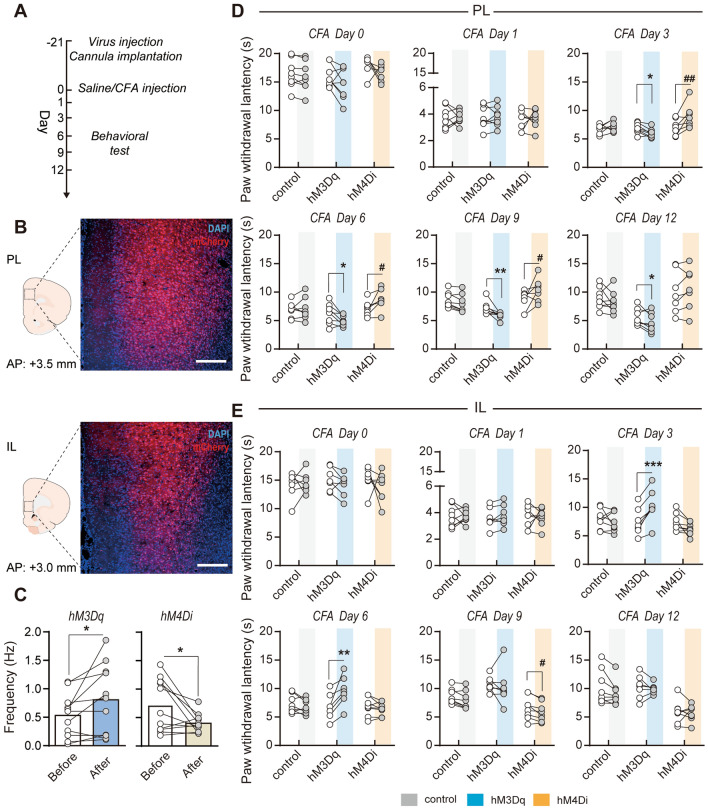
Manipulating PL and IL pyramidal neuronal activity with DREADD-modulated thermal hyperalgesia in rats with inflammatory pain. **A** Diagram showing the experimental timeline. **B** Representative immunofluorescence images showing the expression of the DREADD virus in the PL (upper panel) and the IL (lower panel). Scale bars, 500 μm. **C** Increased/decreased mean firing rate of neurons following administration of CNO after hM3Dq/hM4Di expression. *n* = 11 units in Dq group, *n* = 11 units in Di group. *P <0.05, Wilcoxon signed-rank test. **D** Chemogenetic activation and inhibition of PL significantly exacerbated and attenuated, respectively, CFA-induced thermal hyperalgesia during days 3−12 after modeling. *n* = 7–8 per group. *P <0.05, **P <0.01, Dq *vs* Dq+CNO, ^#^*P* <0.05, ^##^*P* <0.01, Di *vs* Di+CNO, paired *t* test. **E** Chemogenetic activation and inhibition of the IL significantly attenuated and exacerbated, respectively, CFA-induced thermal hyperalgesia during days 3−9 after modeling. *n* = 7–8 per group. **P <0.01, ***P <0.001, Dq *vs* Dq+CNO, ^#^*P* <0.05, Di *vs* Di+CNO, paired *t* test.

To causally evaluate how PL/IL neuronal activity contributed to thermal hyperalgesia, the Designer Receptor Exclusively Activated by Designer Drugs (DREADD) technique was used to achieve temporal- and neuronal type-specific manipulations in the PL and IL (Fig. 4A). We transfected PL/IL pyramidal neurons with a virus containing hM3Dq or hM4Di (Fig. 4B), with local CNO infusion to activate or inhibit local pyramidal neurons, respectively (Dq: *P* = 0.0244; Di: *P* = 0.0322, Wilcoxon signed-rank test; Fig. 4C). CNO was infused at different time points after CFA injection to find when the PL and IL participated in the recovery process from thermal hyperalgesia. Neither manipulation affected the baseline PWLs or thermal hyperalgesia in acute inflammatory pain. PL modulation did not show temporal-specific effects: activation of PL pyramidal neurons consistently aggravated thermal hyperalgesia (CFA days 3–12, Dq group, day 3: *t*_(7)_ = 3.956, *P* = 0.0055; day 6: *t*_(7)_ = 2.470, *P* = 0.0428; day 9:* t*_(7)_ = 2.892, *P* = 0.0234; day 12: *t*_(7)_ = 4.013, *P* = 0.0051, paired *t* test; Fig. 4D), whereas CNO inhibition alleviated thermal hyperalgesia (CFA days 3−9, Di group, day 3: *t*_(6)_ = 2.813, *P* = 0.0306; day 6: *t*_(6)_ = 2.656, *P* = 0.0377; day 9:* t*_(6)_ = 2.480, *P* = 0.0478, paired *t* test; Fig. 4D). However, IL modulation showed more temporal-specific effects: CNO activation of IL pyramidal neurons transfected with AAV-CaMKII-hM3Dq-mCherry induced remarkable analgesia only in the sub-acute stage after CFA injection (CFA days 3–6, Dq group, day 3: *t*_(6)_ = 6.776, *P* <0.001; day 6: *t*_(6)_ = 4.011, *P* = 0.007, paired *t* test; Fig. 4E), but induced slight hyperalgesia in the chronic stage (CFA day 9, Di group, *t*_(7)_ = 3.354, *P* = 0.0122, paired *t* test; Fig. 4E). Similar to lesions, these chemogenetic manipulations did not affect the PWLs in the contralateral paws or in control rats with intraplantar saline injection (data not shown).

Together, these findings support the conclusion that the PL and IL play distinct roles in the alleviation process of thermal hyperalgesia, *i.e.*, PL pyramidal neurons maintain thermal hyperalgesia, whereas IL pyramidal neurons alleviate thermal hyperalgesia in a temporal-specific manner.

### Loss of Drive by the IL Causes Dysfunction of the PL and Thermal Hyperalgesia in Inflammatory Pain

**Fig. 5:**
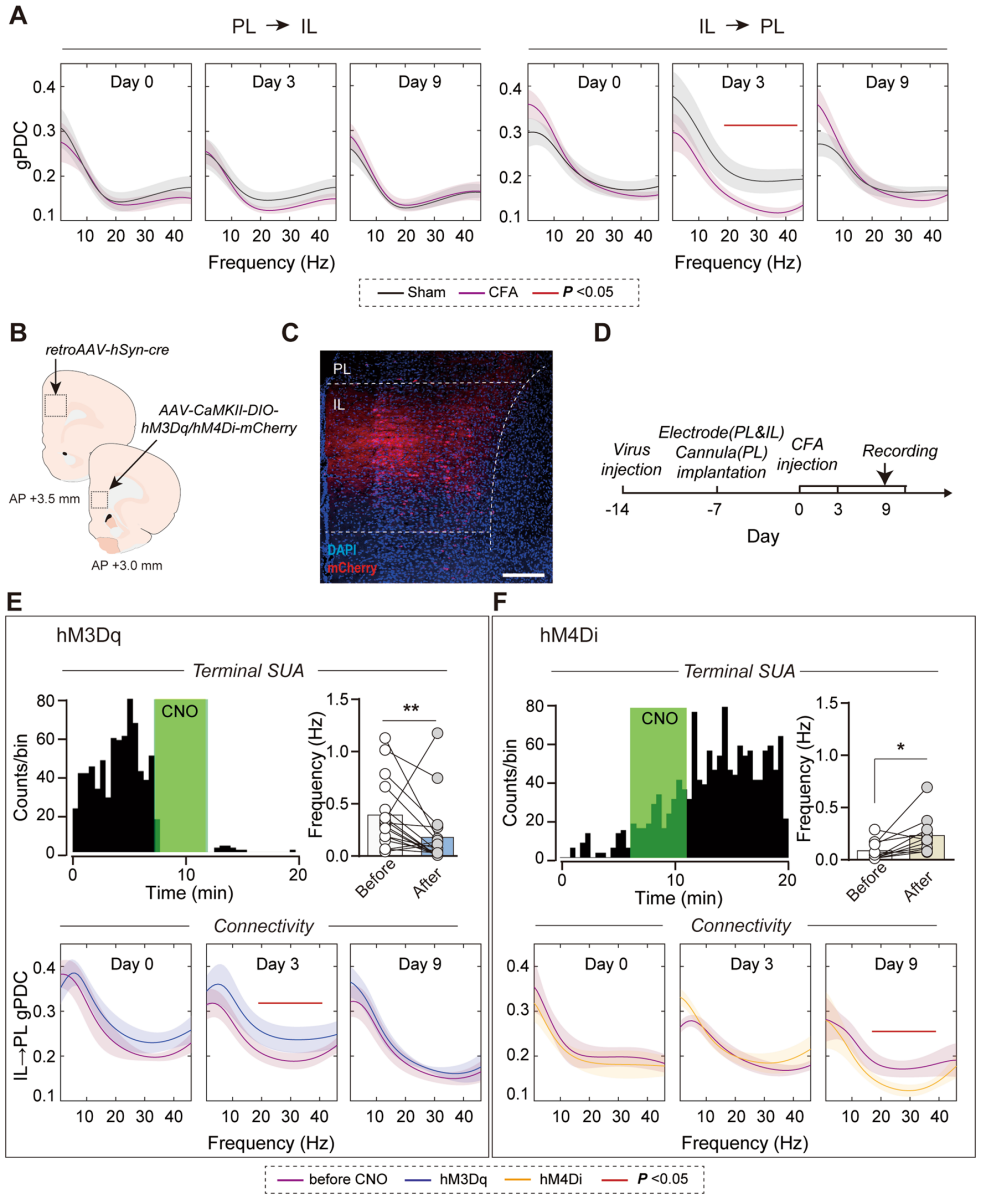
Inhibitory drive from IL to PL is disrupted by peripheral inflammation. **A** Decreased generalized partial directed coherence (gPDC) on day 3 in the CFA group compared to that in the saline control group in the beta and gamma bands (20–46 Hz) from the IL to the PL, but not the opposite direction. Red line, frequency range with statistically significant differences. **B** Schematic of experimental procedure for IL-to-PL labeling. **C** Expression of mCherry fluorescence in IL somata and axon terminals in deep layers of the PL. Scale bar, 500 μm. **D** Diagram showing the recording procedure. **E** A representative *in vivo* rate histogram plot showing inhibited activity of a putative pyramidal neuron in the PL upon CNO application in the vicinity of hM3Dq-expressing IL terminals. Chemogenetic activation of the IL–PL pathway decreases the mean firing rates in the PL and reverses the impaired gPDC from IL to PL on CFA day 3. Red line, the frequency range with statistically significant differences. *n* = 11 units. *P <0.05, Wilcoxon signed-rank test. **F** A representative *in vivo* rate histogram plot showing enhanced activity of a putative pyramidal neuron in the PL upon CNO application in the vicinity of hM4Di-expressing IL terminals. Chemogenetic inhibition of the IL–PL pathway increases the mean firing rates in the PL and reduces gPDC from IL to PL on CFA day 9. Red line, the frequency range with statistically significant differences. *n* = 17 units. *P <0.05, Wilcoxon signed-rank test.

The ipsilateral IL and PL are reciprocally connected [23]. The parallel hypo-functional IL and hyper-responsive PL activities in response to peripheral nociceptive stimuli in inflammatory pain suggest a possible inhibitory drive of the IL over the PL, which projection has been shown to play an important role in associative learning [15]. To test this hypothesis, we analyzed the directional functional connectivity between IL and PL, which was quantified by gPDC based on LFPs. The directional gPDC level from IL to PL decreased in the beta and gamma bands (20–46 Hz) on CFA day 3 but reversed on CFA day 9. By contrast, no significant changes were found in the opposite direction (from PL to IL) (Fig. 5A), which indicated that the modulation from IL to PL was lowered in the early stage.

To investigate the mechanisms underlying possible pathways from IL to PL in pain modulation, we determined whether these adjacent prefrontal areas were directly connected. We delivered a Cre-expressing retrograde AAV specifically to the PL and AAV expressing Cre-dependent DREADD and mCherry for visualization and manipulation of dually-infected neurons in the IL (Fig. 5B). A subset of neurons was detected in the IL, which were projecting to the PL (Fig. 5C). Then *in vivo,* multi-channel recordings were made to verify the functional reliability of the system and explore the electrophysiological features (Fig. 5D). After hM3Dq expression in the IL and the vicinity of the PL, CNO injection into the PL inhibited the firing of PL pyramidal neurons and enhanced IL–PL connectivity on CFA day 3 (firing rate: *P* = 0.0093, Wilcoxon signed-rank test; Fig. 5E), consistent with the reported inhibitory drive of PL excitatory neurons over IL pyramidal cells *via* local interneurons [14]. As expected, hM4Di expression showed increased PL pyramidal neuron firing rates and decreased gPDC from IL to PL on CFA day 9 (firing rate: *P* = 0.0137, Wilcoxon signed-rank test; Fig. 5F). These results indicate that the activity of the IL suppresses the hyper-activity of the PL which contributes to the hyperalgesia in chronic pain.

**Fig. 6:**
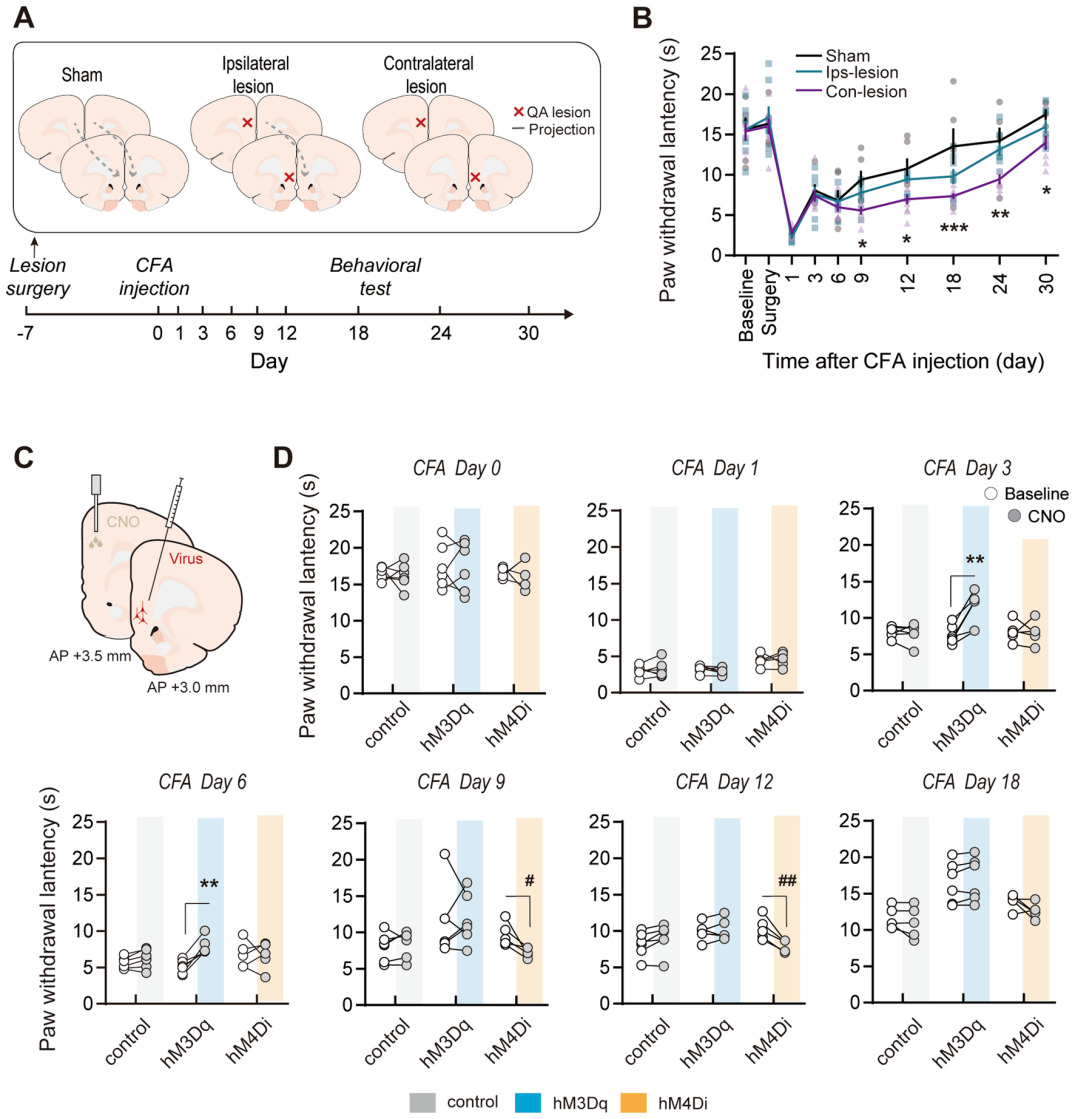
Manipulation of the IL–PL pathway affects inflammatory pain. **A** Schematic diagram of IL–PL disconnection lesions in rats. **B** Disconnection lesions of the IL and the PL prolong the recovery from inflammatory pain. *n* = 6–8 per group. *P <0.05, **P <0.01, ***P <0.001, Con-lesion *vs* Sham. Two-way ANOVA with repeated measures and Bonferroni’s *post hoc* test. **C** Schematic of the procedure of chemogenetic manipulation of the IL-to-PL pathway. **D** CNO activation of the IL–PL pathway attenuates thermal hyperalgesia in the sub-acute phase of inflammatory pain (CFA days 3–6), whereas CNO inhibition of the IL–PL pathway aggravates thermal hyperalgesia in the later phase (CFA days 9–12). *n* = 5–6 per group. **P <0.01, Dq *vs* Dq+CNO, ^#^*P* <0.05, ^##^*P* <0.05, Di *vs* Di+CNO, paired *t* test.

Behaviorally, a disconnection lesion experiment of the IL and the PL was performed (Fig. 6A). Compared with sham group, contralateral, but not ipsilateral, IL and PL lesions induced prolonged thermal hyperalgesia after CFA injection (time effect: *F*_(9,171)_ = 102.8, *P* <0.001; group effect: *F*_(2,171)_ = 4.865, *P* = 0.0197; interaction: *F*_(18,171)_ = 2.218, *P* = 0.0045, two-way ANOVA with repeated measures and Bonferroni’s *post hoc* test; Fig. 6B). This result was similar to IL lesions (Fig. 3E), and indicated an inhibitory modulatory effect of the IL on the PL in inflammatory pain. To test whether the IL–PL pathway modulates inflammatory pain as expected, we used DREADDs to manipulate the pathway directly. We transfected IL pyramidal neurons with a virus containing hM3Dq or hM4Di. CNO delivery in the PL activated or inhibited IL axon terminals (Fig. 6C). Similar to IL manipulations (Fig. 4E), neither activation nor inhibition of the IL–PL pathway affected physiological PWLs or thermal hyperalgesia in the acute stage of inflammatory pain (CFA day 1). Activation of the IL–PL pathway on CFA days 3–6 (Dq group, day 3: *t*_(5)_ = 4.432, *P* = 0.0068; day 6: *t*_(5)_ = 6.040, *P* = 0.0018, paired *t* test; Fig. 6D), but not on later days, attenuated thermal hyperalgesia, whereas inhibition of this pathway aggravated thermal hyperalgesia on CFA days 9–12 (Di group, day 9: *t*_(4)_ = 3.185, *P* = 0.0334; day 12: *t*_(4)_ = 5.102, *P* = 0.0069, paired *t* test; Fig. 6D), but not on earlier days. These findings further confirm our hypothesis that IL inhibitory modulation of the PL controls the thermal hyperalgesia alleviation process.

## Discussion

In the present study, we found functional changes and contributions of two closely interacting sub-regions of the PFC in the development and maintenance of hyperalgesia during inflammatory pain. Our results suggest that PL activity maintains thermal hyperalgesia, whereas IL activity alleviates thermal hyperalgesia in inflammatory pain. More specifically, while the PL exerts a consistent influence on thermal hyperalgesia throughout the development of chronic inflammatory pain, the IL has its effects more dynamically: temporal hypo-function in the sub-acute phase of chronic inflammatory pain (CFA days 3–6), but recovered in the later phase (CFA day 9).

### PL and IL Exert Dichotomous Functions in Chronic Pain

The PL and IL are two major medial prefrontal cortex areas in rodents and are thought to exert dichotomous functions in various processes such as fear memory [24, 25], addiction [26, 27], and stress [28]. More recently, pyramidal neurons in the PL have been reported to be associated with pain-related behaviors [5, 11, 29–31]. We found high evoked responses of PL pyramidal neurons that contribute to maintaining thermal hyperalgesia in inflammatory pain. These results are consistent with a previous *in vitro* study on the hyperactivity of pyramidal neurons of the PL [32]. Intriguingly, the PL did not show a significant difference in the spontaneous neuronal activity in the present study. The PL is not only involved in the encoding of nociceptive information [11] but is also a vital part of the descending inhibitory pathway of pain [33], which participates in analgesia [29, 34]. How the PL spontaneous activity changes in chronic pain varies across studies. However, some studies suggest that the activity of the PL is suppressed in chronic pain [29]. Recent work has identified a distinct nociceptive neuronal ensemble in the PL, whose activities are increased in inflammatory pain [11]. These results indicate that different neuronal subpopulations with unique physiological and functional features compose the PL. These subsets might show different changes in chronic pain. In the present study, we calculated the averaged firing rates of all the recorded putative pyramidal neurons as the spontaneous firing rates. These neurons contained populations of neurons with different changes (activated or inhibited) and overall did not show any evident changes in the spontaneous neuronal firing rates.

Compared with the PL, the IL has been much less investigated in pain. Our previous study has revealed that disrupted connectivity from ventral hippocampus CA1 (vCA1) to the IL leads to spontaneous pain and pain chronicity in rats with inflammation [13]. Evoked nociceptive responses with vCA1–IL activation are attenuated, but only in the period when spontaneous pain is prominent. In this work, we showed temporal-specific effects of chemogenetic control in the IL on pain. Activation of the IL produced marked analgesic effects during 3–9 days after CFA injection. The effective window is consistent with the period of vCA1–IL dysfunction. These results support the role of IL in the regulation of evoked pain. Other studies have reported similar findings of IL hypo-activity in inflammatory pain models [7, 17]. Taken together, neuronal tuning and behavioral studies have suggested the opposite functions of prefrontal sub-regions in chronic inflammatory pain development, implying a potential similarity between pain chronification and other behaviors, like fear and addiction.

### IL Mediates Hyperalgesia Alleviation via Its Inhibitory Regulation of the PL

IL pyramidal neurons inhibit PL outputs [14]. This projection may directly promote the learning of alternative associations [15]. In the present study, both disconnection lesion experiments and electrophysiological results indicated dysfunctional IL–PL connectivity in inflammatory pain. Another intriguing finding was the accelerated recovery from thermal hyperalgesia with an early enhancement of IL–PL projections. The sub-regional interactions in the PFC have been intensively studied in fear learning and extinction [35, 36].

It is worth noting that the neural circuits underlying the IL modulation of pain could involve brain areas other than the PL. The majority of deep-layer prefrontal pyramidal neurons project to and regulate subcortical output systems [37]. The IL inhibits amygdala activation through its projections to GABAergic neurons [38–40]. Depressed IL activity reduces inhibition of the amygdala, which contains nociceptive neurons [41]. In addition, the IL also acts on the ventrolateral periaqueductal gray, which plays a crucial role in the descending control of pain [42].

### An Assumption of Pain Chronification: Impaired Extinction

Our results showed dynamic changes in IL/PL function and IL–PL functional connectivity during the development of inflammatory pain. A rational model fitting these findings would be a temporal inhibition of IL pyramidal neurons in acute inflammatory pain with the most severe thermal hyperalgesia, whose reversal initiates or contributes to recovery from the hyperalgesia. The spontaneous recovery at a later stage (CFA day 9) from the hypo-function of the IL recorded as a lower firing rate and beta-gamma oscillations at the sub-acute stage (CFA day 3) support this model. It is noted that this recovery occurred at the later stage of CFA when thermal hyperalgesia was still present. This anticipated change indicates the causality between these functions and spontaneous hyperalgesia alleviation, which can be supported by the positive relationship between IL beta-gamma oscillation and the recovery rate of CFA.

How to interpret these changes? We regard the pain chronification as an impaired extinction process. Chronic pain has been suggested to be “maladaptive learning” [43, 44]. Findings from our present study provide direct support for this hypothesis: chronic inflammatory pain can be understood as an “extinction” deficit from acute pain. Indeed, contextual and cue fear conditioning depresses IL excitability in rodents [45] and ventromedial PFC functions in humans [46], and lesioning or inactivating the IL prevents fear extinction [47, 48]. Patients with post-traumatic stress disorder show a hypo-functional ventromedial PFC that correlates with their failure to discriminate between safe and dangerous contexts [49]. These results imply some similarities between the memory process and pain chronification. Several hypotheses have pointed out that the chronic pain development model could be interpreted by learning and memory [43, 44, 50]. Our results support the hypothesis that the spontaneous recovery can be regarded as an “extinction process” driven by the IL and thus a loss of IL inhibitory regulation of the PL, which is similar to their functions in fear learning and drug addiction.

### Limitations

Although we confirmed that the IL pyramidal output inhibits PL pyramidal neurons firing, there also exists a GABAergic projection from IL neuropeptide Y-positive interneurons to PL pyramidal neurons in the superficial layer [51]. Furthermore, the changes in the prefrontal microcircuit and the roles of different types of neurons need to be confirmed in chronic pain. Another limitation is that we just focused on thermal hyperalgesia induced by chronic inflammatory pain, but the PFC also participates in pain-induced emotional and cognitive behaviors. These are also interesting questions for the future.

In summary, our results suggest a significant role of the IL and its inhibitory regulation of the PL in the development and maintenance of thermal hyperalgesia in chronic inflammatory pain.

## Data Availability

The datasets and code are available from the corresponding author on request.
